# The Systematic Comparison of Enhancer of Zeste Homolog‐2‐, Bromodomain‐containing Proteins‐, Histone Deacetylase‐, and DNA‐methyltransferase 1‐inhibitors in a Syngeneic Murine Model of Melanoma Reveals Differential Anti‐tumoral and Immunomodulatory Activities

**DOI:** 10.1002/mco2.70336

**Published:** 2025-08-09

**Authors:** Valentina Rigo, Adriana Amaro, Francesco Reggiani, Daniela Fenoglio, Stefania Martini, Tiziana Altosole, Mariangela Petito, Cecilia Profumo, Michela Croce

**Affiliations:** ^1^ UO Biotherapies IRCCS Ospedale Policlinico San Martino Genoa Italy; ^2^ SSD Regulation of Gene Expression IRCCS Ospedale Policlinico San Martino Genoa Italy; ^3^ UO of Experimental Pathology and Immunology IRCCS Ospedale Policlinico San Martino Genoa Italy

1

Dear Editor,

Aberrant epigenetic regulation often occurs in cancer, especially in regulating proliferation, invasion, metastasis, and metabolic reprogramming [1]. Much effort has been dedicated to developing drugs targeting epigenetics as anticancer therapy. Various classes of epigenetic drugs (i.e., inhibitors of Histone Deacetylase: HDAC, Enhancer of Zeste Homolog‐2: EZH2, Bromodomain‐containing Proteins: BET, and DNA‐methyltransferase 1: DNMT) have been tested on primary melanoma cells. The DNMT‐inhibitor (‐i) guadecitabine appeared to most potently modulate the expression of genes related to immune responses [2]; indeed, we recently demonstrated that it can potentiate anti‐PD‐1 and anti‐CTLA‐4 antibodies and reduce the formation of metastases in a melanoma model, by increasing antigen presentation, T‐cell responses, and interferon (IFN)γ production [3]. These data confirm that epigenetic drugs can be excellent add‐ons for cancer immune therapy. Deciphering single epigenetic drug effects in the tumor microenvironment (TME) may help identify the best drug to combine with immune checkpoint inhibitors (ICIs). Therefore, we provide a comparative analysis of inhibitors targeting EZH2 (GSK‐126), BET (OTX‐015), HDAC (vorinostat), and DNMT (guadecitabine) in a syngeneic murine melanoma model, shedding light on their different anti‐tumor and immunomodulatory effects. Vorinostat, OTX‐015, and guadecitabine decreased in vivo B16F10 tumor growth in C57black6J mice (Figure 1A, and Supporting Information), OTX‐015 being the most effective at each measurement time (days 11, 13, and 15). Guadecitabine was slightly efficient at controlling tumor growth; nonetheless, a significant reduction was detected at day 10 post‐tumor cell injection, and, when administered later for 5 consecutive days (5–10 setting), it still induced a significant reduction at day 15 (i.e., 5 days after the end of treatment [EOT]). GSK‐126 had no effects in vivo and only minor effects on the TME. The effects of Vorinostat on TME appear contradictory: it strikingly reduced myeloid‐derived suppressive cells (MDSCs), probably by mediating MDSC apoptosis or contrasting their recruitment [4], but on the other hand, it hampers anti‐tumor recognition by decreasing dendritic cells (DC), thus impairing antigen presentation to adaptive immunity, considering the unique role of DC [5] (Figure 1B), and supported tumor immune escape by up‐regulating PD‐L1 and TIM‐3 expression on B16F10 cells (data not shown). Also, it diminished systemic IL15 levels, thus potentially reducing the activation, proliferation, and survival of T and natural killer cells [6] (Figure 1C). Gene expression analysis of tumors showed that vorinostat differentially expressed 272 genes (241 up‐ and 31 down‐regulated). Gene Set Enrichment Analysis (GSEA) revealed that genes differentially expressed upon vorinostat treatment were enriched for pathways like migration, cell motility, vasculature formation, and angiogenesis (Figure 1D), reflecting changes in the TME. The effects of OTX‐015 and guadecitabine on TME were examined at 2, 7, or 10 days after EOT: OTX‐015 decreased major histocompatibility complex (MHC)‐class I and increased PD‐L1 expression, and guadecitabine up‐regulated MHC‐class I and II, potentially enhancing immunorecognition without increasing immune checkpoints (data not shown) on cancer cells. Although OTX‐015 mainly decreased CD3+ T cells, and at 7 days after EOT, it reduced CTLA‐4+CD39+ regulatory T (Treg) cells, increasing the CD8+/CTLA‐4+CD39+ Treg cells ratio, suggesting prolonged anti‐tumor activity, this is counteracted by a rise in granulocytic‐MDSC levels (Figure 1B). Also, OTX‐015 induced a systemic increase in angiogenic activities (down‐regulating MIG and IP10) and a decrease in Thelper‐2 cytokines (Figure 1C). OTX‐015 produced a significant differential expression of 22 genes: increased TAA, such as *Pmel* and *S100*, but diminished antigen‐presenting machinery, pro‐survival, and anti‐apoptotic gene expression. Significant differences were observed when tumors were excised 7 days post‐treatment, suggesting that OTX‐015 effects are sustained over time. These results indicate that OTX‐015 modulates the TME, reducing the chemoattraction of proinflammatory cells and promoting apoptosis, possibly in tumor cells. Indeed, OTX‐015 has been attributed to inhibit inflammatory signaling pathways, regulate the cell cycle, and induce apoptosis [7]. Guadecitabine increased CD3+ T cells and granzyme‐producing CD8+ and CD4+ T cells. Ten days after EOT, the percentages of PD‐1+CD4+ and PD‐1+CD8+ cells were decreased compared to the control. Also, a significant decrease in CD4+CD25+FoxP3+ Treg cells co‐expressing CTLA‐4 and CD39 immune regulatory molecules [8], suggestive of a shift to immune responsive TME, was associated with a substantial increase in the ratio of CD8+/Treg and CD8+/CTLA‐4+CD39+Treg cells (Figure 1B). Finally, monocytic‐ and granulocytic‐MDSC were depleted, particularly at 2 days after EOT, with a significant increase in the ratio CD8+/MDSC at 10 days after EOT. The increase of systemic G‐CSF, GM‐CSF, and IL6, cytokines involved in MDSC generation, is probably due to a compensatory mechanism following MDSC depletion (Figure 1C). Guadecitabine treatment modified the expression of 794 genes (442 up‐ and 352 down‐regulated) (Figure 1D). Notable differences in gene expression were observed in tumors excised 2 days after EOT, whereas at 10 days after EOT, tumors showed an intermediate expression profile. GSEA of the up‐regulated genes identified cytokine‐cytokine receptor interaction and the JAK/STAT signaling pathway as the most significantly enriched pathways. Among the differentially expressed genes, those related to IFN‐stimulated genes, MHC, and TAA were notably up‐regulated, particularly 2 days after EOT. Interestingly, the guadecitabine‐induced gene expression changes appear transient, as most returned to baseline levels following drug discontinuation. This observation suggests that continuous or cyclic administration of guadecitabine may be necessary to maintain its therapeutic effects.

**FIGURE 1:**
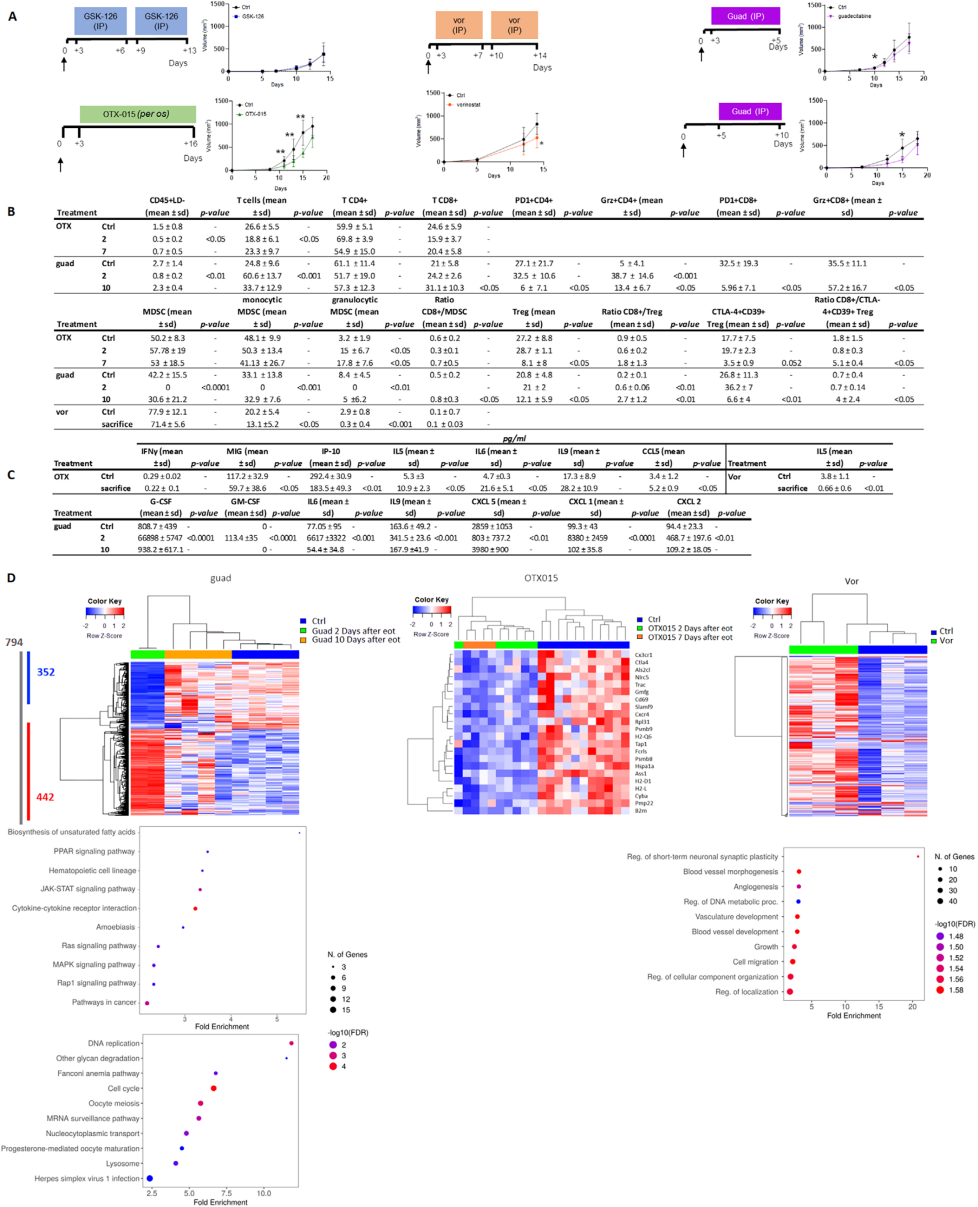
(A) In vivo activity of epigenetic drugs. Schedule of treatments and tumor volume growth in control (Ctrl) and mice treated with GSK‐126, vorinostat (vor), OTX‐015 (OTX), and guadecitabine (guad). *N* = 10 mice/group * *p <* 0.05, ** *p <* 0.01, *** *p <* 0.001. (B) Table embedded in the Figure shows the epigenetic drug effects on TME. Cell suspensions from control and treated tumors were analyzed by flow cytometry. Mean ± SD of percentages and p are indicated. Classical Treg are defined as CD45+CD3+CD4+CD25+Foxp3+; monocytic‐MDSC as Ly6C+Ly6G‐/CD11b+, and granulocytic‐MDSC as Ly6ClowLy6G+/CD11b+, referred to as CD11b+CD45+ cells. *N* = 8 mice/group have been analyzed * *p <* 0.05, ** *p <* 0.01, *** *p <* 0.001, **** *p <* 0.0001. (C) Table embedded in the Figure shows cytokine or chemokine modifications induced by epigenetic drug treatments. Serum cytokine or chemokine levels expressed in pg/mL from mice treated with vorinostat, OTX‐015, and guadecitabine. *N* = 8–10 mice/group data are presented as mean ± sd * *p <* 0.05, ** *p <* 0.01, *** *p <* 0.001, **** *p <* 0.0001. (D) Epigenetic drug treatment affects gene expression. Top panel (left to right): Heatmap of differentially expressed genes in tumors from: Ctrl (blue line) and guadecitabine‐treated removed 2 (green line) and 10 (light brown line) days after EOT; OTX‐015‐treated mice, collected at 2 (green line) and 7 (light brown line) days after EOT; vorinostat‐treated mice (green line). Sample size: *n* = 2–10 mice/group. **Middle panel: Left**: KEGG pathway enrichment analysis of 442 genes up‐modulated by guadecitabine, **Right**: Gene Ontology (GO) analysis of biological process affected in vorinostat‐treated tumors compared to controls. **Bottom panel**: KEGG pathway enrichment analysis of 352 down‐modulated genes by guadecitabine. Bubble plots: the horizontal axis indicates fold enrichment (Ratio of differentially expressed genes present in the GO or KEGG pathways to the total number of genes in the reference gene set), the vertical axis shows the categories from the Gene Ontology Biological Process or KEGG pathways.

Our analysis indicates that epigenetic alterations of chromatin structure can modulate the tumor immune response by restoring the expression of components of the antigen‐presenting machinery and reducing the presence of immunosuppressive cells. Selecting epigenetic drugs for combination with ICI requires careful consideration of their impact on the TME. Not all epigenetic agents enhance ICI efficacy; some may hinder its therapeutic effects. Our data demonstrate that guadecitabine induces modifications on both tumor and immune cells, making the former more recognizable by immune cells and the latter more active, by decreasing MDSC and Treg and increasing the ratio CD8/Treg, thus being the most suitable among epigenetic drugs tested, to be associated with immunotherapy. Indeed, a phase 2 clinical trial exploits this therapeutic combination in patients with melanoma and non‐small cell lung cancer  (NIBIT‐ML1 and NCT04250246). Finally, this study contributes valuable insights into potential treatment strategies for melanoma and highlights the importance of understanding the immunomodulatory properties of epigenetic drugs in cancer therapy.

## Author Contributions

**V.R**.: In vivo experiments, methodology, statistical analysis, and data curation. **A.A**.: Molecular biology experiments, methodology, statistical analysis, and data curation. **F.R**.: Methodology, statistical analysis, and data curation. **D.F**.: Flow cytometry analysis, formal analysis, visualization, and data curation. **S.M**.: In vitro multiplex ELISA experiments. **T.A**.: Flow cytometry analysis and data curation. **M.P**.: Molecular biology experiments. **C.P**.: Flow cytometry analysis and methodology. **M.C**.: Conceptualization, study design, data interpretation, original draft writing, review, and editing. All authors have read and approved the final manuscript.

## Ethics Statement

Animal studies were approved by the IRCCS Ospedale Policlinico San Martino of Genova ethics committee (OPBA) and authorized by the Italian Ministry of Health (n°783/2018‐PR released on October 15, 2018, according to art.31 legislative decree 26/2014).

## Consent

All authors have approved the submitted version (and any substantially modified version that involves the author's contribution to the study).

## Conflicts of Interest

The authors declare no conflicts of interest.

## Supporting information




**Supporting File 1**: mco270336‐sup‐0001‐SuppMat.docx

## Data Availability

Microarrays and RNA sequencing data are available under accession numbers GSE254073 and GSE254070, respectively (http://www.ncbi.nlm.nih.gov/geo/).
