# Supplementary material for: The Systematic Comparison of Enhancer of Zeste Homolog‐2‐, Bromodomain‐containing Proteins‐, Histone Deacetylase‐, and DNA‐methyltransferase 1‐inhibitors in a Syngeneic Murine Model of Melanoma Reveals Differential Anti‐tumoral and Immunomodulatory Activities
Source: MedComm (2020). 2025 Aug 9;6(8):e70336. doi: 10.1002/mco2.70336 (PMC12334842; doi:10.1002/mco2.70336)
Supplement: Supplementary file 1 — Supporting File 1: mco270336‐sup‐0001‐SuppMat.docx [file MCO2-6-e70336-s001.docx]

**Supplementary Information for**

**The systematic comparison of Enhancer of Zeste Homolog-2-, Bromodomain-containing proteins-, Histone Deacetylase-, and DNA-methyltransferase 1-inhibitors in a syngeneic murine model of melanoma reveals differential anti-tumoral and immunomodulatory activities**

**Running title: Immunomodulatory Effects of Epigenetic Drugs**

Valentina Rigo^1,#^, Adriana Amaro^2,#^, Francesco Reggiani^2^, Daniela Fenoglio^1^, Stefania Martini^3^, Tiziana Altosole^1^, Mariangela Petito^2^, Cecilia Profumo^1^, and Michela Croce^1,*^

^1^ UO Biotherapies, IRCCS Ospedale Policlinico San Martino, Genova, Italy. ^2^ SSD Regulation of gene expression, IRCCS Ospedale Policlinico San Martino, Genova, Italy. ^3^UO of Experimental Pathology and Immunology, IRCCS Ospedale Policlinico San Martino, Genova, Italy.

^#^ equally contributed to the work

*correspondence to: [michela.croce@hsanmartino.it](mailto:michela.croce@hsanmartino.it)

Materials and Methods

Cell lines and reagents: B16F10 (ATL99010) mouse melanoma cell line was purchased from ICLC (Genova, Italy; authentication by institutional biological banking facility using STR according to International Cell Line Authentication Committee (ICLAC) guidelines)^1^.

Animal model: Eight-week-old C57black6J female mice (Charles River Laboratories Italia Srl) were housed in individually ventilated cages (IVC) at the Animal Facility of the IRCCS Ospedale Policlinico San Martino of Genova with 12-h dark/light cycles after approval by the IRCCS Ospedale Policlinico San Martino of Genova ethics committee (OPBA) and authorization by the Italian Ministry of Health (n°783/2018-PR released on 15/10/2018, according to art.31 legislative decree 26/2014). All procedures on animals were performed according to the National and European guidelines for the care and use of laboratory animals (EEC directive 276/33/2010 and D.L. 26/2014). Mice were injected subcutaneously (SC) in the right flank with 10^5^ B16F10 (>90% viable) in a volume of 0.1ml serum-free medium. Mice were monitored for disease symptoms every other day, manually. Endpoints for sacrifice were a tumor volume reaching 1 cm^3^ or the appearance of signs of disease or suffering. Tumor volume calculation: V= ½ x L x W x H.

Tumor-bearing mice were treated with: EZH2 histone-lysine N-methyltransferase inhibitor, GSK-126, (adapted from^2^); HDAC inhibitor vorinostat, (adapted from^3^); BET and bromodomain inhibitor OTX-015, (adapted from^4^); and DNMT inhibitor guadecitabine (adapted from^5^). Mice were sacrificed by CO_2_ asphyxiation, and at the sacrifice, tumors, and blood were taken from the treated and controls. Tumors were used for immunofluorescence, gene expression analyses, and blood for cytokine/chemokine detection.


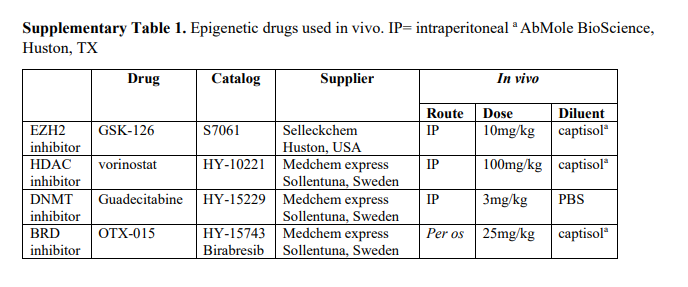


Immunofluorescence analysis: Antibodies used, catalog number, the supplier, and gating strategies were previously described^1^. The samples were analyzed by a BD Fortessa X20 flow cytometer (BD Biosciences) using the BD FACS Diva™ software version 8.0 (BD Biosciences) or Flowjo (Ashland, USA).

MILLIPLEX Multiplex ELISA: Serum from blood samples, collected from treated and control mice at sacrifice were analyzed by Milliplex Map kit^1^, using a Luminex MagPix reader with xPONENT software (Millipore).

Microarrays and RNA sequencing analyses: RNA was extracted from the tumors using the RNeasy Plus mini kit (Qiagen, Hilden, Germany). RNA quality was assessed with Nanodrop and BioAnalyzer tools (Agilent, St. Clara, CA). cDNA, ds-cDNA, and cRNA synthesis and fragmentation were performed using the 3′ IVT Express Kit (Affymetrix, Santa Clara, CA, USA). Hybridization, washing, and staining were performed using the GeneAtlas® (Affymetrix, St. Clara, CA) following the manufacturer’s instructions. Microarrays were preprocessed in R/BioConductor^6,7^. Library preparation and sequencing Universal Plus Total RNA-Seq with NuQuant kit (Tecan Genomics, Redwood City, CA) has been used for library preparation following the manufacturer’s instructions (library type: fr-secondstrand). RNA samples were quantified and quality tested by Agilent 2100 Bioanalyzer RNA assay (Agilent Technologies, Santa Clara, CA) or Caliper (PerkinElmer, Waltham, MA). Final libraries were checked with both Qubit 2.0 Fluorometer (Invitrogen, Carlsbad, CA) and Agilent Bioanalyzer DNA assay or Caliper (PerkinElmer, Waltham, MA). Libraries were then prepared for sequencing and sequenced on paired-end 150 bp mode on NovaSeq6000 (Illumina, San Diego, CA). The dataset is available under accession number GSE254073.

RNA-Seq data as FastQ files were processed with the nf-core/rna-seq pipeline^8,9^. Briefly raw data files (FastQ) quality was assessed with FastQC [https://www.bioinformatics.babraham.ac.uk/projects/fastqc/], low-quality bases and adapter sequences were removed with Trim Galore [https://www.bioinformatics.babraham.ac.uk/projects/trim_galore/], in the final step data was aligned with Salmon^10^ to produce a matrix file with genes as rows and samples as columns, the number of reads aligned on each gene were reported as transcripts per million (tpm). The dataset is available under accession number GSE254070.

Statistical analyses: All the cytofluorimetric comparisons between control and treated mice were analyzed by two-sided T-test for independent samples or two-way or one-way ANOVA for different groups of treatments and control. P values lower than 0.05 were considered significant: *p<0.05, **p<0.02, ***p<0.01, ****p<0.001. Statistical analyses were performed using PRISM 9.4 (Graph-Pad Software, San Diego, CA, USA).

References:

1. Amaro A, Reggiani F, Fenoglio D, et al. Guadecitabine increases response to combined anti-CTLA-4 and anti-PD-1 treatment in mouse melanoma in vivo by controlling T-cells, myeloid derived suppressor and NK cells. *J Exp Clin Cancer Res*. 2023;42(1):67. doi:10.1186/s13046-023-02628-x

2. Chaudhary P, Guragain D, Chang JH, Kim JA. TPH1 and 5-HT7 Receptor Overexpression Leading to Gemcitabine-Resistance Requires Non-Canonical Permissive Action of EZH2 in Pancreatic Ductal Adenocarcinoma. *Cancers (Basel)*. 2021;13(21):5305. doi:10.3390/cancers13215305

3. Wang L, Leite de Oliveira R, Huijberts S, et al. An Acquired Vulnerability of Drug-Resistant Melanoma with Therapeutic Potential. *Cell*. 2018;173(6):1413-1425.e14. doi:10.1016/j.cell.2018.04.012

4. Berenguer-Daizé C, Astorgues-Xerri L, Odore E, et al. OTX015 (MK-8628), a novel BET inhibitor, displays in vitro and in vivo antitumor effects alone and in combination with conventional therapies in glioblastoma models. *Int J Cancer*. 2016;139(9):2047-2055. doi:10.1002/ijc.30256

5. Luo N, Nixon MJ, Gonzalez-Ericsson PI, et al. DNA methyltransferase inhibition upregulates MHC-I to potentiate cytotoxic T lymphocyte responses in breast cancer. *Nat Commun*. 2018;9:248. doi:10.1038/s41467-017-02630-w

6. Irizarry RA, Hobbs B, Collin F, et al. Exploration, normalization, and summaries of high density oligonucleotide array probe level data. *Biostatistics*. 2003;4(2):249-264. doi:10.1093/biostatistics/4.2.249

7. Mirisola V, Mora R, Esposito AI, et al. A prognostic multigene classifier for squamous cell carcinomas of the larynx. *Cancer Lett*. 2011;307(1):37-46. doi:10.1016/j.canlet.2011.03.013

8. Ewels P, Hammarén R, Peltzer A, et al. *Nf-Core/Rnaseq: Nf-Core/Rnaseq Version 1.4 “Gray Crocus Dachshund.”* Zenodo; 2019. doi:10.5281/zenodo.3490660

9. The nf-core framework for community-curated bioinformatics pipelines | Nature Biotechnology. Accessed January 15, 2024. https://www.nature.com/articles/s41587-020-0439-x

10. Patro R, Duggal G, Love MI, Irizarry RA, Kingsford C. Salmon provides fast and bias-aware quantification of transcript expression. *Nat Methods*. 2017;14(4):417-419. doi:10.1038/nmeth.4197
